# RETRACTION: Effect of General and Spinal Anaesthesia on Post‐Operative Wound Healing During Laparoscopic Totally Extraperitoneal Inguinal Hernia Repair: A Meta‐Analysis

**DOI:** 10.1111/iwj.70396

**Published:** 2025-03-21

## Abstract

**RETRACTION**: KongL. , WangJ. , and GuoK. , “Effect of General and Spinal Anaesthesia on Post‐Operative Wound Healing During Laparoscopic Totally Extraperitoneal Inguinal Hernia Repair: A Meta‐Analysis,” International Wound Journal 21, no. 2 (2024): e14670, 10.1111/iwj.14670.38361225 PMC10869659

The above article, published online on 15 February 2024, in Wiley Online Library (http://onlinelibrary.wiley.com/), has been retracted by agreement between the journal Editor in Chief, Professor Keith Harding; and John Wiley & Sons Ltd. Following an investigation by the publisher, all parties have concluded that this article was accepted solely on the basis of a compromised peer review process. The editors have therefore decided to retract the article. The authors did not respond to our notice regarding the retraction.

**RETRACTION**: L. Kong , J. Wang , and K. Guo , “Effect of General and Spinal Anaesthesia on Post‐Operative Wound Healing During Laparoscopic Totally Extraperitoneal Inguinal Hernia Repair: A Meta‐Analysis,” International Wound Journal 21, no. 2 (2024): e14670, 10.1111/iwj.14670.38361225 PMC10869659

The above article, published online on 15 February 2024, in Wiley Online Library (http://onlinelibrary.wiley.com/), has been retracted by agreement between the journal Editor in Chief, Professor Keith Harding; and John Wiley & Sons Ltd. Following an investigation by the publisher, all parties have concluded that this article was accepted solely on the basis of a compromised peer review process. The editors have therefore decided to retract the article. The authors did not respond to our notice regarding the retraction.

